# The Effect of the Platelet Administration for the Patients with Liver Dysfunction after Liver Resection: Preliminary Clinical Trial

**DOI:** 10.1155/2021/9948854

**Published:** 2021-09-08

**Authors:** Hui Xu, Yu-Mei Li, Yongxiang Yi, Yun-Wen Zheng, Nobuhiro Ohkohchi

**Affiliations:** ^1^Institute of Regenerative Medicine and Affiliated Hospital of Jiangsu University, Jiangsu University, Zhenjiang 212001, Jiangsu, China; ^2^Department of Surgery, The Second Hospital of Nanjing, Nanjing 210003, China; ^3^Department of Gastrointestinal and Hepato-Biliary-Pancreatic Surgery, Faculty of Medicine, University of Tsukuba, Tsukuba 305-8575, Japan; ^4^Guangdong Provincial Key Laboratory of Large Animal Models for Biomedicine, and School of Biotechnology and Heath Sciences, Wuyi University, Jiangmen 529020, Guangdong Province, China; ^5^Yokohama City University School of Medicine, Yokohama, Kanagawa 234-0006, Japan

## Abstract

The aim of this study is to investigate the effect of platelet on the improvement of deteriorated liver function after liver resection. Six patients with hepatocellular carcinoma and liver cirrhosis have received the partial hepatectomy in the institution. Their Child–Pugh grade was B, and platelet count was below 7,000/*µ*l. After hepatectomy, 20 units of platelet transfusion were carried out, liver function and side effects were investigated after 4 weeks, and the number of platelets increased to approximately 15,000/*µ*l. Liver functions, such as aspartate transaminase (AST), alanine aminotransferase (ALT), cholinesterase (ChE), and prothrombin time, as well as albumin, recover to the same level as those before operation and 4 weeks after the operation. Any side effects were not recognized in all patients. Administration of platelets for cirrhotic patient with hepatectomy was carried with safety. But remarkable effect on the improvement of liver function was not recognized.

## 1. Introduction

Hepatocellular carcinoma (HCC) is one of the most common cancers in the world [1]. The estimated new HCC cases and deaths of China was 466 × 10^3^ and 422 × 10^3^ every year, respectively [2]. The decrease of the platelet number was frequently observed with abnormal liver function and cirrhosis in the process of HCC. The conditions make the liver resection dangerous with the high risk of bleeding, hepatic failure, infection, and disseminated intravascular coagulation (DIC) [3, 4].

Alkozai reported that low count of platelet was associated with delayed liver function recovery after hepatectomy [5]. There are same reports in which platelets promote the liver regeneration after liver resection and prevent hepatocytes deterioration [6–8]. In addition, platelets also suppress and improve liver fibrosis [9, 10]. Therefore, we expected an improvement of liver function after liver resection by administration of platelet-rich plasma in the perioperative period. In clinical research, we assessed the safety and efficiency of platelet transfusion after hepatectomy in patients with HCC and with decreased number of platelets in the perioperative period.

## 2. Materials and Methods

Six patients with HCC and liver cirrhosis, i.e., Child–Pugh B, received hepatectomy at the Department of General Surgery in the Affiliated Hospital of Jiangsu University and the Second Hospital of Nanjing from November 2016 to March 2017 (Table 1). Age, operation type of hepatectomy, duration, and blood loss of the operation were described in Table 1. HCC was diagnosed by computed tomography (CT) scan and biopsy. They received 20 units of platelet-rich plasma transfusions 48 hours after hepatectomy. One patient received additional 20 units of platelet transfusion one week after operation. Patient characteristics are described in Table 1. This study was permitted by the ethical committee of both institutes. Informed consent was obtained from all individual participants. The experimental clinical trial was approved by the institutional board of the hospital review board and performed in accordance with the Declaration of Helsinki (clinical trial no. ChiCTR1800019523). Inclusion criteria were as follows: age from 20 to 70 (years); primary HCC; Child–Pugh grade B; platelet count from 20 to 70 (×10^9 ^L); and chronic hepatitis (cirrhosis). Exclusion criteria were as follows: acute hepatitis; metastatic hepatic carcinoma; HIV infection; and severe brain and circulation system disease.

### 2.1. Outcome Parameters and Laboratory Variables

We made comprehensive evaluation of general condition and liver function before and after the surgery. We carried out blood test for liver function, i.e., albumin (alb), AST, ALT, total bilirubin (T-Bil), direct bilirubin (D-Bil), cholinesterase (ChE), and fibrotic marker, i.e., hyaluronidase (HA), laminin (LN), type IV collagen (CIV), procollagen III *N*-terminal propeptide (PIIINP), at the time of before, 72 h, and 1 month after liver resection. Postoperative complication was also investigated.

### 2.2. Statistical Analysis

Results are presented as mean ± standard deviation. Data in the three groups of before, 72 hours, and 1 month after operation were statistically analysed using the chi-squared test or Fisher's exact test. Statistical analysis was done using *T*-test.

## 3. Results

### 3.1. Postoperative Platelet Numbers and Postoperative Liver Function Values

Pre- and postoperative platelet number, values of liver function, and PT and liver fibrosis markers are indicated in Table 2. The platelet number at the time of 72 h after operation was significantly higher than that of preoperative (*P*=0.047), and there was no difference of platelet number between before operation and 1 month after operation. The ChE level at the time of 72 h after operation was a little higher than before operation (*P*=0.322). The D-Bil level at the time of 1 month after operation is also a little higher than before operation (*P*=0.008). There was no significant difference between liver fibrosis marker at the time of 1 month after operation and before operation.

### 3.2. Postoperative Complications

One patient had bile leakage but improved after 6 days. There were no other side effects by platelet transfusion.

## 4. Discussion

Surgeries including liver resection and liver transplantation are the most effective treatments for liver cancer patients who could achieve long-term survival. With the hypersplenism in the process of liver cirrhosis, the platelet number of patients often decreases. This condition would increase the risk of liver resection. The phenomenon of decrease in peripheral platelet counts is frequent after hepatectomy [11]. Therefore, it is necessary to maintain the level of platelet count in perioperative and postoperative period [12].

Platelet has the special effect for the liver such as promotion of liver regeneration, prevention of liver damage, and improvement of liver fibrosis [7, 9, 13]. Murata et al. reported three pathways of hepatocyte proliferation by platelets [14]. Hisakura et al. reported that platelets suppress the activation of caspase 3. Therefore, the apoptosis of sinusoidal lining cells and hepatocyte induced acute hepatitis in the liver damage [8]. Ikeda et al. reported that platelets have the suppressive effect on the hepatic stellate cells, i.e., hepatic fibroblast, through adenosine and cyclic AMP [15]. In our study with the transfusion of platelet, the platelet number increased approximately to 15,000/*µ*l at 72 h after surgery. The platelet life span is 3 to 4 days, and the platelet number decreased to the same level of the preoperative condition after 1 month. On the other hand, in patients with normal liver, the number of platelets increased 1 month after surgery [12, 16]. The reason of the different phenomenon is due to the degree of deterioration of the liver function. The ChE level in 72 h after operation is a little higher than the preoperative values, and the reason is supposed that 10 units of platelet-rich plasma contained the plasma of 200–300 ml, and the plasma includes Alb, ChE, and cytokine.

In summary, we evaluated the platelet administration effects on the improvement of the liver function in the patients with liver dysfunction after hepatectomy. The results indicated that the safety of the large amount transfusion of platelets was clarified. However, the improvement of liver function was not recognized.

## Figures and Tables

**Table 1:** Patient characteristics of 6 patients.

Age (years old)	58.5 ± 6.24
Sex	Male: 6; female: 0
Child–Pugh score	7.66 ± 1.21
Number of resected liver segments	
≤2	3
3	2
4	1
Hospital stay (days)	15.7 ± 3.3
Viral infection	HBV (*n* = 5), HCV (*n* = 1)
AFP (ng/ml)	44.4 ± 57.9
Blood loss during operation (ml)	131.2 ± 55.6

Note: values are presented mean ± standard deviation. AFP, alpha-fetoprotein.

**Table 2:** Blood test of preoperative and postoperative of liver resection in 6 patients.

Laboratory variable (normal value)	Preoperative	Postoperative	
		72 h	1 month
PLT (100–300 × 10^9^)/L	39.2 ± 9.3	51. ± 10.3^*∗*^	42.8 ± 12.5
AST (5–40) (U/L)	38.8 ± 20.6	35 ± 10.5	39.5 ± 15.1
ALT (5–40) (U/L)	44.5 ± 13.2	39.3 ± 19.5	42.5 ± 20.9
ALP (40–129) (U/L)	115 ± 55.3	95.2 ± 31.7	83.5 ± 20.8
PT (9–13) (sec)	18.8 ± 3.5	17.7 ± 2.4	17.2 ± 3.4
Alb (35–55) (g/ml)	28.4 ± 2.9	27.7 ± 2.1	29 ± 1.9
T-Bil (0–12) (*µ*mol/L)	16.4 ± 5.5	14.1 ± 4.3	11.9 ± 3.8^＃^
D-Bil (0.5–6.8) (*µ*mol/L)	9.1 ± 2.6	9.1 ± 5.7	5.3 ± 1.2
LDH (109–245) (U/L)	204.3 ± 49.6	177.7 ± 43.9	171.3 ± 39.4
ChE (4230–13000) (U/L)	2196 ± 556	254 ± 590 (*P*=0.32)	2211 ± 420
HA (0–120) (ng/ml)	322 ± 308		304 ± 268
LN (0–130) (ng/ml)	65 ± 43.4		68.7 ± 31.7
CIV (0–95) (ng/ml)	73.5 ± 36.9		71.3 ± 28.1
PIIINP (0–15) (ng/ml)	13.5 ± 6.8		13 ± 5.4

Note: values are presented mean + standard deviation.72 h versus preoperative platelet ^*∗*^*P*=0.047, 1 month versus preoperative D-Bil ^＃^*P*=0.008. PLT, platelet; AST, aspartate transaminase; ALT, alanine aminotransferase; ALP, alkaline phosphatase; PT, prothrombin time; Alb, albumin; T-Bil, total bilirubin; D-Bil, direct bilirubin; LDH, lactate dehydrogenase; ChE, cholinesterase; HA, hyaluronidase; LN, laminin; CIV, type IV collagen; PIIINP, procollagen III *N*-terminal propeptide.

## Data Availability

All of the data used in the article can be found in the medical record database of the Second Hospital of Nanjing.

## Abbreviations

PLT: Platelet
AST: Aspartate transaminase
ALT: Alanine aminotransferase
ALP: Alkaline phosphatase
PT: Prothrombin time
Alb: Albumin
T-Bil: Total bilirubin
D-Bil: Direct bilirubin
LDH: Lactate dehydrogenase
ChE: Cholinesterase
HA: Hyaluronidase
LN: Laminin
CIV: Type IV collagen
PIIINP: Procollagen III *N*-terminal propeptide
Akt: Anaplastic lymphoma kinase
Erk: Extracellular regulated protein kinases
IL-6: Interleukin-6
TNF-*α*.: Tumour necrosis factor-*α*.
